# Simultaneous Detection of Polar and Nonpolar Molecules by Nano-ESI MS with Plasma Ignited by an Ozone Generator Power Supply

**DOI:** 10.3390/molecules30122546

**Published:** 2025-06-11

**Authors:** Yunshuo Tian, Yifan Meng, Richard N. Zare

**Affiliations:** 1 College of Letter and Science, University of California, Santa Barbara, CA 93106, USA; 2 Department of Chemistry, Stanford University, Stanford, CA 94305, USA

**Keywords:** ozone generator power supply, dielectric barrier discharge ionization, nonpolar molecules, mass spectrometry

## Abstract

We present a simple and cost-effective method for generating a dielectric barrier discharge (DBD) plasma using a commercially available ozone generator power supply. By coupling the plasma source to the extended ion transfer tube of an ambient mass spectrometer, we achieved stable plasma discharge, enabling the post-ionization of nonpolar compounds during the electrospray ionization process. Using this approach, we successfully detected polycyclic aromatic hydrocarbons (PAHs), halogenated PAHs (HPAHs), and other nonpolar pollutants in liquid mixtures, with detection limits on the order of 10 ng/mL. In fish exposed to HPAHs, both polar metabolites and lipids, as well as the nonpolar pollutant 1-chloronaphthalene, were simultaneously detected. Notably, 1-chloronaphthalene accumulated at the highest concentration in gill tissue. This straightforward plasma-assisted technique offers a reliable strategy for expanding the detection capabilities of electrospray mass spectrometry to include nonpolar molecules.

## 1. Introduction

Mass spectrometry (MS) is a powerful analytical tool for characterizing a wide range of chemical compounds, owing to its high sensitivity and specificity [1]. Among various ionization techniques, electrospray ionization (ESI) has become a key method for liquid-phase analysis, particularly for polar compounds [2,3,4]. For example, ESI has been successfully used in metabolomics [5,6,7,8], lipidomics [9,10,11], and environmental studies [12,13,14]. However, conventional ESI faces significant challenges in ionizing nonpolar or low-polarity molecules, such as polycyclic aromatic hydrocarbons (PAHs) [15] and their halogenated derivatives (HPAHs) [16], which lack functional groups that can be easily protonated or deprotonated. This limitation restricts the application of ESI-MS in environmental monitoring and exposomics [17], where both hydrophobic pollutants and endogenous metabolites coexist.

To address these challenges, several ambient ionization strategies have been developed, including atmospheric pressure chemical ionization (APCI) [18] and atmospheric pressure photoionization (APPI) [19,20]. Although effective, these methods often require gas flows, high-cost equipment, or UV sources, which increase system complexity and operational costs. Plasma-based techniques have recently emerged as promising alternatives for efficiently ionizing nonpolar or low-polarity molecules that are difficult to analyze by conventional ESI-MS. For instance, the combination of laser-induced plasma and ESI-MS has been shown to be a powerful approach for detecting multiple nonpolar compounds [21,22,23,24]. Similarly, low-temperature plasma (LTP) generated via dielectric barrier discharge (DBD) has been widely used for the soft ionization of nonpolar species, producing intact molecular ions with minimal fragmentation [25,26,27,28]. However, the generation of DBD typically requires a high-voltage, high-frequency alternating current (AC) power supply, which is not commonly available in most laboratories, potentially limiting the broader application of this technique.

In this work, we present a plasma-assisted nanoESI method based on dielectric barrier discharge ionization (DBDI), powered by a compact, simple, and low-cost ozone generator power supply. By coupling this discharge source to a standard nanoESI interface, we generated a localized low-temperature plasma at the ion transfer region under ambient conditions. This configuration enabled the direct ionization of nonpolar molecules that are difficult to detect using conventional ESI, while retaining the ability to ionize polar compounds from the same sample. We have demonstrated the utility of this system through the detection of representative PAHs and HPAHs in methanolic solutions, as well as the simultaneous detection of low-polarity pollutants and endogenous lipids in fish tissue extracts. The results show that this method significantly broadens the chemical coverage of nano ESI-MS and provides a versatile tool for the simultaneous analysis of structurally diverse molecules in complex systems. Its compact design, low cost, and dual-mode ionization capability make it highly suitable for a wide range of environmental and biological applications.

## 2. Results

### 2.1. DBDI Source Powered by an Ozone Generator Power Supply

**Figure 1:**
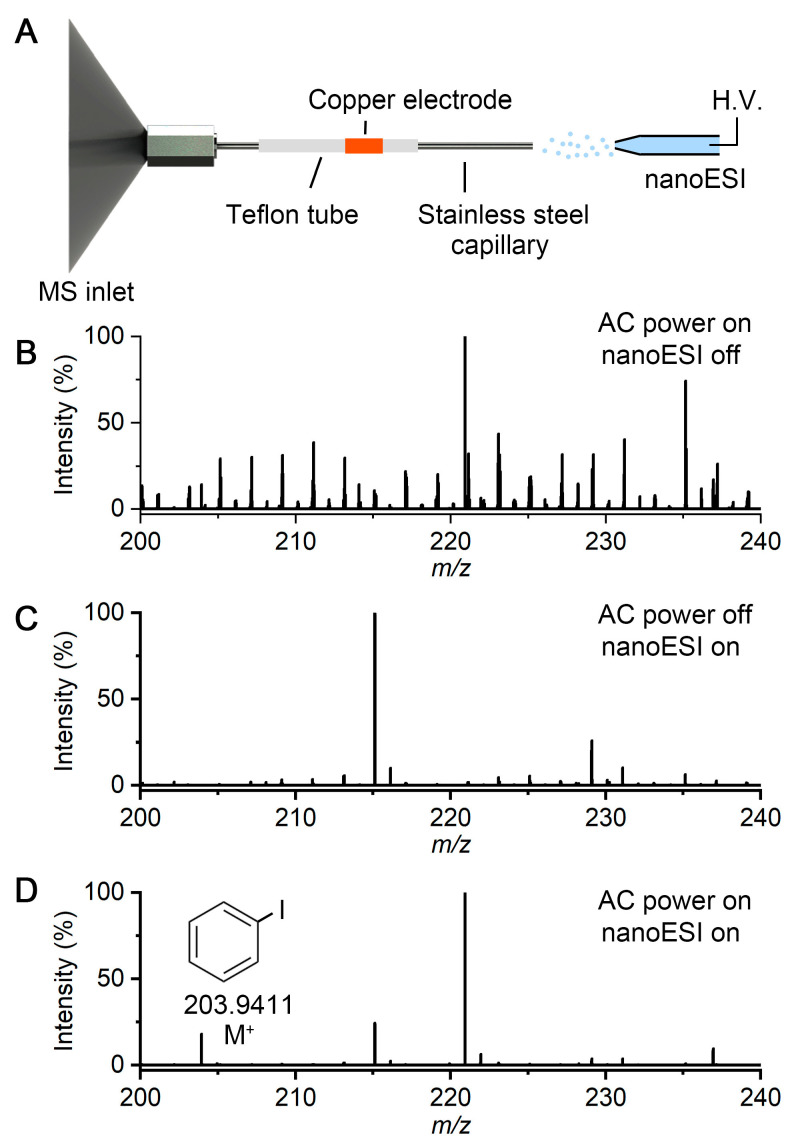
Ionization of low-polarity compound in nanoelectrospray by the discharge ignited through an ozone generator power supply. (**A**) Schematic diagram of the experimental setup. (**B**) Mass spectrum of air plasma. (**C**) Mass spectrum of the nanoelectrosprayed sample when turning off the plasma. (**D**) Mass spectrum of chemical components in the nanoelectrospray when turning on the plasma. Iodobenzene can be detected at *m*/*z* 203.9411.

Figure 1A shows the scheme of the DBDI source, which was inspired by the previous “active sampling capillary” configuration [26]. Briefly, a Teflon tube (i.d. 1/16 inch and o.d. 1/8 inch), which serves as the dielectric, was coupled to the ion transfer tube (with 1 cm extension) of the mass spectrometer (Orbitrap Velos Pro, Thermo Fishier, San Jose, CA, USA). The pressure gradient between the atmospheric environment and the vacuum inside the mass spectrometer facilitates a constant airflow of 1.6 L/min through the ion transfer tube. A stainless-steel capillary (i.d. 0.5 mm and o.d.1/16 inch) inserted into the Teflon tube served as the grounded electrode. Meanwhile, a copper ring (i.d. 1/8 inch) wrapped tightly around the Teflon tube served as the H.V. electrode. The nano ESI source is used as the sample injection setup. A heating tube is used to set the temperature of the stainless-steel capillary to 350 °C to achieve high-efficiency solvent evaporation. The copper ring and the stainless-steel capillary are connected to the ozone generator power supply (input: DC 12 V, output: AC 5 kV, 50 kHz), which was from a commercial compact ozone generator.

When the ozone generator power supply is turned off, this setup serves as a conventional nano ESI ionization source. When the AC power supply is turned on, low-temperature plasma is generated inside the Teflon tube by DBD and ionizes the nonpolar molecules from the sample solution, which is difficult for nano ESI. As a proof of concept, 10 μM iodobenzene was prepared in a mixture of water and acetonitrile (volume 1:1) and injected into the mass spectrometer from the nano ESI capillary. Figure 1B shows the mass spectrum of the air background when the ozone generator power supply was turned on. When we turned off the AC voltage and injected solution into the mass spectrometer, iodobenzene cannot be detected by nano ESI for the reason of its low polarity (Figure 1C). Figure 1D shows the mass spectrum of chemical components in the solution when switching on the ozone generator during nano ESI injection. It takes about 10 s for the ozone generator power supply to achieve a stable plasma. An iodobenzene peak (M^+^) can be detected at a *m*/*z* of 203.9411. This result shows that this ozone generator power supply is effective for generating a discharge to enhance the ionization efficiency of low-polarity compounds when using electrospray ionization.

The improved detection of nonpolar molecules in our DBDI-nanoESI setup can be attributed to two plasma effects. First, DBD in air produces long-lived metastable species (e.g., O_2_^+^ and N_2_^+^) that undergo Penning ionization or charge transfer with analyte molecules, generating intact molecular ions without requiring protonation sites. It is also possible that the localized high electric field at the nanoESI emitter enhances field-induced ionization of highly polarizable species. By combining these pathways, the plasma source efficiently liberates and ionizes hydrophobic contaminants that are otherwise invisible to conventional ESI.

### 2.2. Detection of Multiple PAHs and HPAHs in Liquid Sample

To evaluate the capability of the nanoESI-DBDI setup for detecting low-polarity compounds, a mixture of six PAHs was prepared. In brief, naphthalene, acenaphthene, pyrene, benzo[k]fluoranthene, and benzo[ghi]perylene were dissolved in the mix solution of methylene chloride and methanol (1:1). Each of the concentrations is 1 ppm. Sample introduction was performed using a pulled quartz tube emitter with a spray voltage of 1.6 kV applied to the emitter tip. AC plasma was continuously active during data acquisition, while no sheath or auxiliary gas was used.

**Figure 2:**
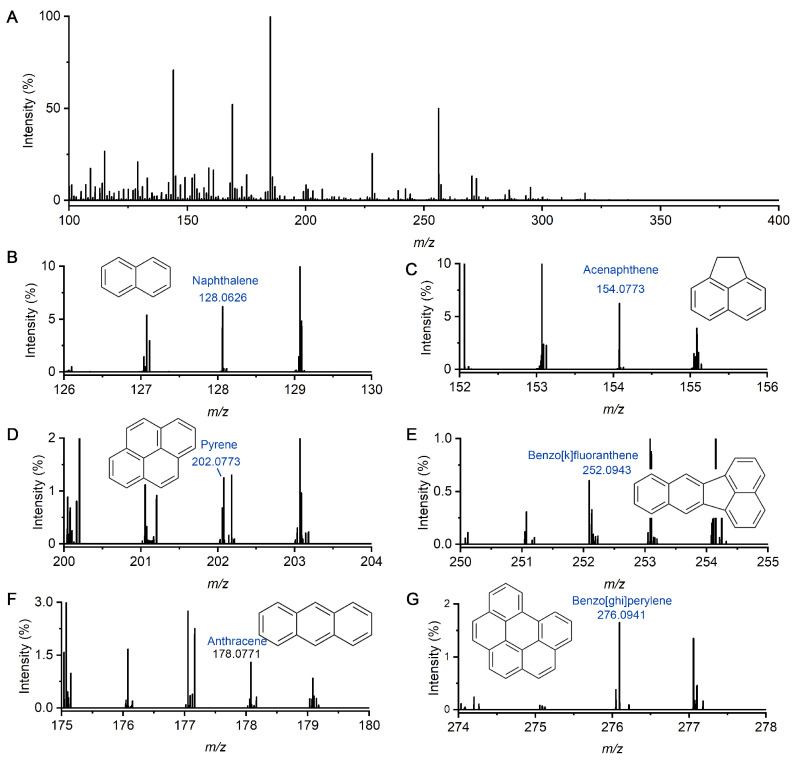
Detection of PAHs mixture in a solution of methylene chloride and methonal (1:1). (**A**) Full mass spectrum of the solution injected by nanoESI and ionized by the plasma. (**B**–**G**) Mass spectra of naphthalene at *m*/*z* of 128.0626, acenaphthene at *m*/*z* of 154.0733, pyrene at *m*/*z* of 202.0773, benzo[k]fluoranthene at *m*/*z* 252.0943, anthracence at *m*/*z* of 178.0771, and benzo[ghi]perylene at *m*/*z* of 276.0941, respectively.

Figure 2A displays the full mass spectrum of the PAHs solution injected by nanoESI and ionized by the plasma. When plasma was generated in the ion transfer tube, these gas molecules can be detected by the mass spectrometer. As shown in Figure 2B–G, the peak of naphthalene, acenaphthene, pyrene, benzo[k]fluoranthene, anthracene, and benzo[ghi]perylene can be clearly identified at *m*/*z* of 128.0626, 154.0773, 202.0773, 252.0943, 178.0771, and 276.0941. These nonpolar molecules are difficult to protonate and are not detected under conventional nanoESI conditions. In contrast, under plasma activation, molecules can be ionized. The observed mass errors were within ±3 ppm. The full-scan spectra showed low background noise, with signal-to-noise ratios exceeding 100 for most peaks. These results indicate that plasma assistance is efficient for the ionization and MS detection of nonpolar PAHs.

**Figure 3:**
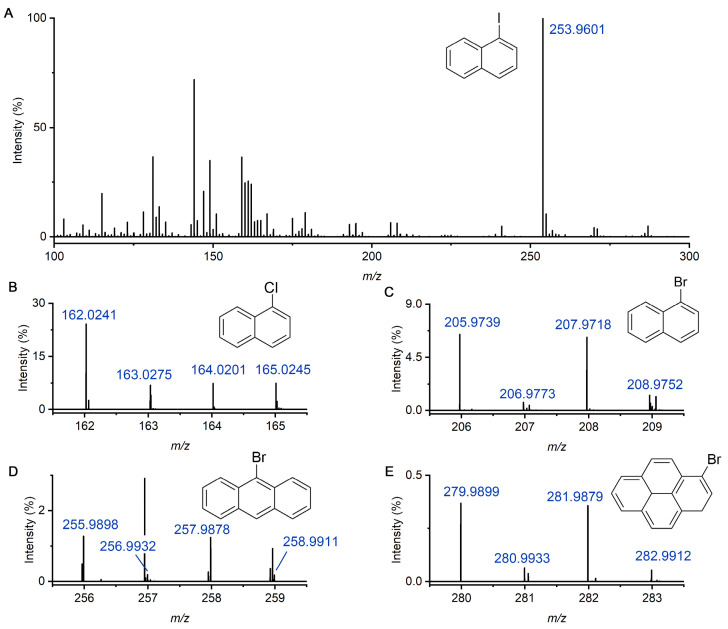
Detection of HPAHs mixture in methanol–methylene chloride solution. (**A**) Full mass spectrum of the solution injected by nanoESI and ionized by the plasma. The peak at *m*/*z* of 253.9601 is identified to be 1-iodonaphthalene. (**B**–**E**) Mass spectra of 1-chloronaphthalene, 1-bromonaphthalene, 9-bromoanthracene, and 1-bromopyrene, respectively.

Similarly, 1-iodonaphthalene, 1-chloronaphthalene, 1-bromonaphthalene, 9-bromoanthracene, and 1-bromopyrene were also mixed in methylene chloride and methanol (1:1). The concentration of each compound was 1 ppm. Figure 3A showed the mass spectrum of the mixture of these HPAHs. Among 1-iodonaphthalene, 1-chloronaphthalene, and 1-bromonaphthalene, 1-iodonaphthalene has the lowest ionization potential (8.03 eV). Therefore, it has the highest signal intensity as shown in the mass spectrum, and the detected *m*/*z* is 253.9601. Figure 3B–E display mass spectra of 1-chloronaphthalene, 1-bromonaphthalene, 9-bromoanthracene, and 1-bromopyrene, respectively. These compounds exhibited distinct molecular ion clusters that closely matched their theoretical isotopic patterns. For example, 1-bromopyrene showed isotope peaks at *m*/*z* of 279.9899, *m*/*z* of 280.9933, *m*/*z* of 281.9879, and *m*/*z* of 282.9912, consistent with natural bromine abundance. Similarly, chlorinated species displayed characteristic doublet peaks with a near 3:1 ^35^Cl/^37^Cl distribution, as expected. The accurate reproduction of these isotopic signatures further supports the reliability and selectivity of the DBDI-based ionization. None of the PAHs or HPAHs yielded detectable signals under nanoESI alone (AC off), confirming that the discharge-generated plasma is essential for the ionization of molecules lacking protonation sites. Across five replicate injections, signal intensities showed relative standard deviations below 20%, demonstrating acceptable repeatability for semi-quantitative analysis.

Compared to traditional APPI or APCI sources that require additional UV lamps or heated gas, the nanoESI DBDI setup by the ozone generator power supply is simpler, more compact, and cost-efficient, yet capable of delivering similar ionization performance for hydrophobic organic analytes. These findings highlight the potential of this approach for the routine screening of environmental pollutants, petroleum-derived compounds, or other nonpolar or low-polar analytes in complex matrices.

### 2.3. Limit of Detection Evaluation

To assess the sensitivity of the nanoESI-DBDI setup, we evaluated the limit of detection (LOD) using 1-chloronaphthalene as a model compound. A series of standard solutions with concentrations ranging from 5 ng/mL to 500 ng/mL were prepared in methanol and injected through the same nanoESI emitter under plasma-on conditions. Chlorobenzene (100 ng/mL) was used as the internal standard. The ratio of signal at *m*/*z* 162.0241 and 112.0071, corresponding to the molecular ion M^+^ of 1-chloronaphthalene and chlorobenzene, was used as the quantification target.

**Figure 4:**
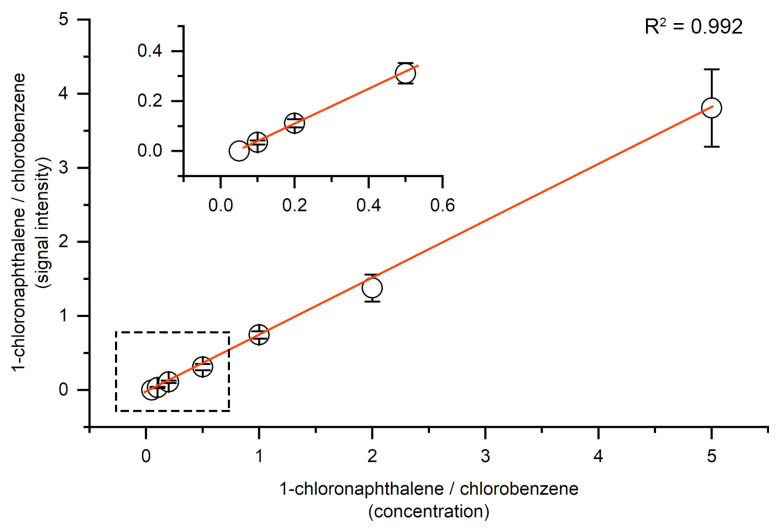
Plot of the signal intensity ratio and concentration ratio of 1-chloronaphthalene/chlorobenzene, showing strong linear behavior. Error bars represent one standard deviation based on three measurements.

Figure 4 shows the signal intensity ratio of 1-chloronaphthalene and chlorobenzene at different 1-chloronaphthalene concentrations. The signal intensity represents the absolute ion counts of the monoisotopic molecular ion [M]^+^ at *m*/*z* 162.0241. The compound produced clear and stable signals down to 10 ng/mL. The calibration curve was based on integrated peak intensities across three replicate injections. The resulting calibration plot demonstrated good linearity over the sample concentration range (10 ng/mL to 500 ng/mL), with a correlation coefficient (R^2^) of 0.992. When the 1-chloronaphthalene concentration is below 10 ng/mL, a stable mass spectrometric signal cannot be obtained. Therefore, the limit of detection was estimated to be approximately 5–10 ng/mL. The relative standard deviation of replicate signals across the linear range remained below 15%, indicating acceptable repeatability for quantitative analysis. Compared to conventional APCI or APPI methods, the DBDI ignited by the ozone generator power supply can achieve a similar detection limit of nonpolar compounds like PAHs [29,30,31,32,33,34,35] (Table 1). We will try to combine this setup with liquid chromatography (LC) or gas chromatography (GC) equipment to further improve the detection ability in future study.

This performance compares favorably with conventional ambient ionization techniques for hydrophobic analytes. Notably, such sensitivity was achieved without derivatization, matrix additives, or dopant gases. The ionization efficiency of 1-chloronaphthalene under nanoESI-DBDI was sufficiently high to enable direct quantification from simple methanol solutions, demonstrating the utility of this method for trace-level analysis of low-polarity contaminants.

### 2.4. Simultaneous Detection of Low-Polarity HPAH and Polar Molecules in Fish

To demonstrate the applicability of this nanoESI-DBDI method to real biological samples, we analyzed tissue extracts obtained from a common goldfish exposed to 1-chloronaphthalene. The experiment aimed to evaluate this approach by simultaneously detecting low-polarity environmental pollutants and endogenous polar biomolecules in complex biological samples. The fish was kept in water containing 10 ppm 1-chloronaphthalene for two hours. After exposure, organs including the gill, liver, intestine, and muscle were harvested and homogenized (10 mg for each organ). Then, 1 mL acetonitrile was used for extraction, and the supernatant was directly analyzed using the same plasma-assisted nanoESI configuration.

**Figure 5:**
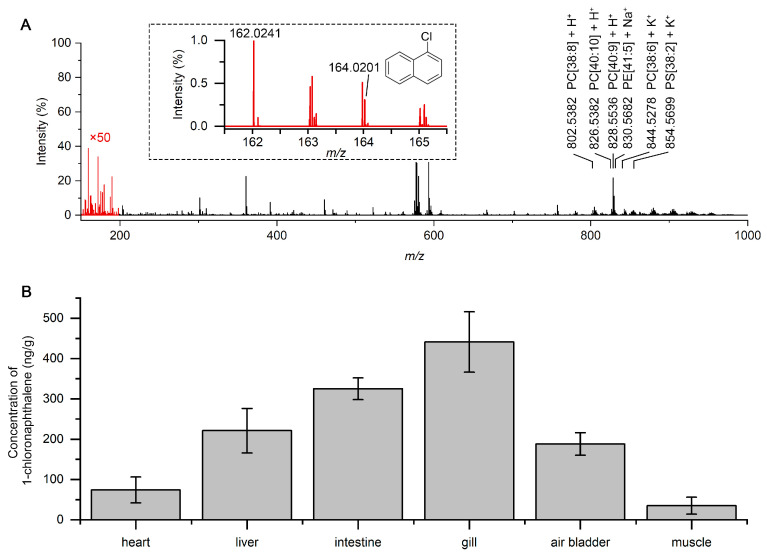
Simultaneous detection of 1-chloronaphthalene and polar lipids in a fish exposed to 1-chloronaphthalene. (**A**) Typical mass spectrum of the intestine extract from a fish. Zoomed-in spectrum shows the nonpolar peak of 1-chloronaphthalene. (**B**) Quantification of 1-chloronaphthalene in different organs from the fish.

Figure 5A shows a typical mass spectrum obtained from the intestinal extract of the fish. The ion signal for 1-chloronaphthalene was observed at *m*/*z* 162.0241, with the isotopic pattern of ^35^Cl/^37^Cl confirming its identity. In addition to the pollutant signal, a series of high-intensity peaks were observed in the *m*/*z* 600–900 range, corresponding to endogenous polar lipids, such as phosphatidylcholines (PCs), lysophospholipids, and other glycerophospholipid species. These polar molecules were efficiently ionized by the nanoESI process. However, 1-chloronaphthalene required plasma activation. The ability to detect both species types within a single scan demonstrates the dual-mode ionization capability of this setup.

The concentration of 1-chloronaphthalene in six organs was examined, and the results are presented in Figure 5B. The liver, air bladder, intestine, and gill have a higher pollutant signal intensity than the heart and muscle. This result is consistent with the expected absorption and distribution behavior of hydrophobic pollutants, which tend to accumulate in organs with high blood perfusion and metabolic activity. The coexistence of low-polarity and polar compounds in the same spectrum allows for comprehensive metabolic and toxicological profiling from minimal sample preparation. Such capability is particularly valuable in environmental toxicology and ecotoxicological biomonitoring, where simultaneous tracking of pollutants and host biochemical responses is desired. This demonstration confirms that the DBDI-nanoESI system not only enhances ionization of low-polarity compounds but also supports comprehensive analysis of biological extracts without sacrificing polar metabolite coverage. The simultaneous detection of both pollutant and endogenous molecules establishes this platform as a powerful tool for environmental exposure assessment and biomolecular response analysis in a single run.

## 3. Discussion

This work demonstrates a low-cost and effective approach for enhancing nanoESI-based mass spectrometry using a dielectric barrier discharge ionization (DBDI) source powered by a compact ozone generator. The plasma-assisted setup enables efficient ionization of low-polarity and nonpolar compounds, which are generally undetectable by conventional ESI because of their lack of protonation sites. The mild plasma conditions produced by the AC-powered discharge are sufficient to generate intact molecular ions with minimal fragmentation, and repeatability across replicates remained within acceptable limits. The evaluation of the limit of detection shows that the quantification ability of the approach for low-polarity molecules can achieve ng/g level. The simultaneous detection of low-polarity pollutants and endogenous polar lipids in fish demonstrates that the method has the potential to monitor multiple molecules of different polarity in a same mass spectrometry scan.

This dual-mode ionization capability makes the method particularly attractive for applications in environmental analysis, exposomics, and biological monitoring, where chemically diverse analytes co-exist in complex systems. For example, in environmental monitoring, this method can simultaneously detect non-polar pollutants (e.g., polycyclic aromatic hydrocarbons, chlorinated organic compounds, persistent organic pollutants) and polar markers, making it ideal for rapid screening of water, soil, or air without the need for complex sample pretreatment or chromatographic separation. In the field of food safety and contaminant analysis, the dual-mode ionization can be used to detect hydrophobic pesticides and lipid-based adulterants in complex matrices. In metabolomics and lipidomics, both endogenous polar metabolites (amino acids, small organic acids) and low-polarity lipids (triglycerides, phospholipids) in biological fluids or cell extracts can be analyzed simultaneously in a single test, simplifying the process and reducing sample consumption. Notably, the DBDI-nanoESI setup may be further adapted to high-throughput platforms by coupling with automated sample injection systems or emitter arrays, enabling sequential or parallel analysis in future developments.

Interestingly, recent studies have shown that a mass spectrometer can achieve on-site detection of hazardous volatile organic compounds in combination with a robot or even a drone [36,37]. Owing to the low power consumption and compact size of our DBDI device, it has significant potential for miniaturization. The ion source can be integrated with field-deployable mass spectrometers (e.g., compact ion traps) to enable the on-site screening of environmental pollutants.

## 4. Materials and Methods

### 4.1. Ionization Source Based on the Ozone Generator Power Supply

A Teflon tube (i.d. 1.1 mm, o.d. 1.55 mm) serves as the dielectric barrier and is directly coupled to the mass spectrometer inlet capillary. A stainless-steel capillary (i.d. 0.5 mm, o.d. 1.05 mm) inserted coaxially into the Teflon tube functions as the grounded electrode, while a 2 mm long copper ring (i.d. 1.6 mm) wrapped around the tube acts as the high-voltage electrode; both electrodes and dielectric share a common axis. By adopting an “active sampling capillary” design, no auxiliary gas is required—ambient air alone serves as carrier. Applying a 50 kHz alternating voltage of 5 kV across the electrodes ignites a dielectric barrier discharge inside the tube, generating plasma that ionizes sample molecules in the airflow. This lab-built interface thus extends the MS inlet for ambient plasma-assisted ionization.

### 4.2. Chemicals

All chemicals were purchased from Sigma Aldrich (St. Louis, MO, USA).

### 4.3. Sample Preparation

#### 4.3.1. PAH and HPAH Standards

Mixed stock solutions of PAHs (naphthalene, acenaphthene, anthracene, pyrene, benzo[k]fluoranthene, benzo[ghi]perylene) and HPAHs (1-iodonaphthalene, 1-chloronaphthalene, 1-bromonaphthalene, 9-bromoanthracene, and 1-bromopyrene) were each prepared at 1 ppm in methylene chloride and methanol (1:1).

#### 4.3.2. Fish Tissue Extraction

Fish exposed to 1-chloronaphthalene were euthanized and organs (heart, air bladder, gill, liver, intestine, muscle) were rapidly rinsed with water. Approximately 10 mg of each tissue was placed in a 1 mL acetonitrile. Samples were homogenized using a bead mill, sonicated for 2 min, and centrifuged at 12,000 rpm for 5 min. The supernatant was transferred to a fresh tube and filtered through a 0.22 µm PTFE membrane.

### 4.4. Safety Considerations

The DBDI-nanoESI setup involves high-voltage AC (5 kV, 50 kHz) and ozone generation in the presence of organic solvents. To mitigate electrical shock and fire risks, all high-voltage components are fully insulated and enclosed within a grounded Faraday cage. Ozone and solvent vapors are removed via a local exhaust hood equipped with activated-carbon filters. Laboratory personnel wear appropriate PPE (nitrile gloves, safety goggles) and continuously monitor ambient ozone levels to ensure concentration remains below recommended exposure limits.

## Figures and Tables

**Table 1:** LOD comparison of this method and other methods for detection of nonpolar compounds.

Analyte	Method	Chromatography	LOD	Reference
PAHs	APLI	Yes	50 pg/mL	[31]
PAHs	APPI	Yes	1 ng/mL	[32]
PAH	APCI	Yes	5 ng/mL	[29]
NPAHs	APCI	Yes	0.4 ng/mL	[33]
PAHs	APCI	Yes	0.7 ng/mL	[34]
PAHs	Laser-induced plasma	No	22 ng/mL	[23]
Pyrimethamine	SPME-DBDI	No	8 ng/mL	[26]
Chlorpyrifos	LTP	No	1 ng/mL	[35]
PAHs & HPAHs	nanoESI-DBDI	No	5–10 ng/mL	This work

## Data Availability

Data are available on reasonable requests from the corresponding authors.
